# Observations on Intermittents

**Published:** 1809-09

**Authors:** 


### *To the Editors of the Medical and Physical Journal.*

GENTLEMEN,

I Should have been much disappointed if you had shut the door indiscriminately against all anonymous communications. The exclusion of severe or petulant criticism from that privilege, appears highly reasonable, and doubtless will be very generally approved; there are, however, two subjects, or occasions, which seem to have a pre-eminent claim to the veil of concealment, which anonymous publication affords. The one is, for the communication of unsuccessful cases; the other for the promulgation of new hints or conjectures respecting the causes or cure of diseases. There is no doubt that the publication of cases, which have baffled the skill of a fair practitioner, tends as much to the improvement of the science as details of successful treatment; but there are many, especially among the junior part of the profession, who would hardly be prevailed on to authenticate their want of success in a public manner, by their signatures. So there are some theoretic conjectures, relating to the causes and cure of diseases, which may appear of small importance to many, or perhaps no better than the offspring of a lively imagination, but which eventually lead to improvements in practice. Under this conviction, I have submitted the following following observations to you, if you deem them worthy of the attention of your Readers.

Intermittents have been unusually prevalent in London during the last spring and summer, and, as I think, unusually obstinate. It appears to have been generally admitted, that an ague cannot originate from any other cause than marsh miasma, or the effluvia issuing from putrescent vegetable and animal substances in marshy and fenny situations. This idea, I believe, has been universally entertained since the opinions of Lancisci on that subject received the sanction of our Cullen. London practitioners, however, begin to grow somewhat sceptical, because they have so often found agues appear in the metropolis during the spring months. I confess, till this spring I never met with a case where the patient had not been previously exposed to marshy situations in the course of some part of the foregoing six months; and I concluded that the contagion had lain dormant till some exciting cause during the cold or rainy weather had called it into action. My opinion on this point, I believe, was far from singular, as I never heard any practitioner express a doubt respecting the marshy origin of intermittents. I have, however, during the present summer, had two instances of ague, where the cause could not be traced to any other than the miasmata of London. In both these patients there was sufficient reason to be convinced of a material derangement in the functions of the liver; and in all the obstinate cases, this derangement was conspicuous in the complexion and albuginea of the eyes, as well as in the colour of the stools and urine. Now, it has long been known that a protracted intermittent seldom fails to injure the liver in a manner similar to the injury produced by hot climates. It is also admitted, that the remittents of hot climates, generally called bilious remittents, approach very nearly to the nature of intermittents; but that the greater fatality attending them, arises from the superior virulence of the exciting cause, when applied to persons unused to the climate. For it has long been acknowledged, that any person in the vigour of life, by a prudent attention to food, drink, rest, and cloathing, may assimilate his constitution to any climate. Other animals have not the same power, because they cannot regulate the non-naturals, unless man, for his own gratification, chooses to take this trouble for them; in which case, elephants, lions, and tigers may be naturalized to our fickle climate. But I am in danger of wandering from the subject of intermittents. If

I have

I have made myself understood in the foregoing observations, it will be credible, if not probable or evident, that the liver demands particular attention in the investigation of the causes and regulation of the cure of ague.

The present season has sent many of the London labourers into Kent, Sussex, and Essex, to assist in getting in the harvest. Hence we may expect a plentiful crop of intermittents to spring up, many of which will be imported into London. After the above introduction, I hope the following queries and hints will not be thought irrelevant.

1. Since intermittents and their causes uniformly derange the functions of the liver, may not a diseased liver strongly predispose to intermittents?

2. Since the habit of labourers in and near London of drinking spirits, has a tendency to derange the functions of the liver, may not this diseased state of that viscus render our labourers much more susceptible of that disease than those who reside in the country?

3. If this view of the subject be correct, may not some preventive means be employed, calculated to obviate this hepatic predisposition?

4. When the disease has supervened, should not the state of the liver be attended to in the first instance? and has any medicine equal powers with mercury to fulfil this indication?

The Author hopes these few observations and hints will draw from some of your numerous and respectable Correspondents, either approbation of, or objections to, his opinions; and for the present subscribes,

HEPATICUS.

*London, August* 16, 1809.

